# Distal Phalanx Fracture of the Toe With Sesamoid Entrapment in the Interphalangeal Joint: A Case Report

**DOI:** 10.1002/ccr3.72221

**Published:** 2026-03-11

**Authors:** Weihao Zhu, Yan Cheng, Xu Sun

**Affiliations:** ^1^ Department of Orthopedics, the Affiliated Taizhou People's Hospital of Nanjing Medical University, Taizhou School of Clinical Medicine Nanjing Medical University Taizhou Jiangsu Province China; ^2^ Taizhou Second People's Hospital Taizhou Jiangsu Province China

**Keywords:** case report, entrapment, foot fracture, phalangeal fracture, sesamoid bone

## Abstract

Phalangeal fractures are common among foot injuries, with the majority amenable to conservative treatment. This case involved a fracture of the toe combined with intra‐articular sesamoid entrapment. To prevent nonunion, surgical intervention was recommended. We present a rare case of distal phalanx fracture of the toe with interphalangeal joint sesamoid entrapment, review the intraoperative challenges encountered, and summarize postoperative insights with recommendations. We report a 41‐year‐old female construction worker who presented with a crush injury to her right great toe. Diagnosis was confirmed radiographically. She underwent closed reduction and internal fixation with Kirschner wire (K‐wire) pinning. Follow‐up examinations showed callus formation at 6 weeks, after which the K‐wire was removed upon fracture healing. Sesamoid entrapment, where the sesamoid becomes lodged within the joint space, typically causes pain, swelling, and restricted motion and is clinically uncommon. Cases of distal phalangeal fractures of the toe complicated with sesamoid entrapment in the interphalangeal joint are rarely reported. There is no consensus on whether to remove the entrapped sesamoid. Preoperatively, we plan to perform an open excision of the sesamoid bone. Combined with the patient's preference and local soft tissue condition, open reduction would cause relatively large trauma to the soft tissues. After discussion, we decided to perform closed reduction of the fracture followed by fixation with a single 1.2‐mm K‐wire. However, intraoperatively, the entrapped sesamoid acted as a lever, preventing anatomical reduction. Given the lower reduction requirements for the distal phalanx, we proceeded with approximate reduction and K‐wire fixation. Phalangeal fracture of the toe with interphalangeal joint sesamoid entrapment is rare. In cases requiring surgery, percutaneous K‐wire fixation can achieve direct short‐term efficacy, but it has long‐term risks and limitations. Based on the practical difficulties encountered, we believe that open reduction combined with sesamoid excision is a better surgical option, providing patients with a more reliable long‐term prognosis.

## Key Clinical Message

Surgical management of toe phalanx fractures with interphalangeal joint sesamoid involvement: experience and recommendations.

## 1 Introduction

Phalangeal fractures are frequent foot injuries, usually resulting from direct (e.g., crush) or indirect (e.g., twist) trauma, presenting with pain, swelling, deformity, and subungual hematoma. Cases complicated by interphalangeal joint sesamoid entrapment are exceptionally rare. Normally, sesamoids do not enter the joint cavity. Traumatic displacement of a sesamoid into the interphalangeal joint is uncommon; to date, reports of congenital entrapment of the sesamoid in the joint space are extremely rare. There is still some controversy and insufficient evidence regarding the clinical treatment and functional prognosis of this condition. Clinical experience is limited concerning the relationship between surgical selection, intraoperative strategies, and postoperative outcomes. Based on the above clinical issues, we retrospectively analyzed this case to explore the optimal surgical strategy for this type of fracture. The patient sustained an injury from a heavy object to the right foot, resulting in pain and swelling of the hallux. Radiographic examination confirmed fractures of the distal and proximal phalanges. Additionally, we identified an aberrant sesamoid bone in the interphalangeal joint, which is not normally present. Radiography of the patient's left foot also revealed a sesamoid within the interphalangeal joint, suggesting possible congenital bilateral entrapment. Due to healthcare policy constraints, CT scans of both feet were not performed. The pathogenesis of sesamoid entrapment is complex, involving multiple factors such as trauma, degeneration, anatomical variation, and biomechanical abnormalities. However, to date, high‐quality clinical evidence supporting the independent existence of congenital sesamoid entrapment remains lacking. Existing relevant studies are deficient in anatomical basis, epidemiological data, imaging control, and long‐term follow‐up. Meanwhile, congenital factors are intertwined with acquired factors such as trauma and degeneration, making it difficult to distinguish them clearly. As no definitive guidelines exist regarding sesamoid excision, and the patient had no prior functional impairment, to avoid negative impacts caused by more operations. We considered not resecting the sesamoid bone, and after evaluating multiple surgical plans, we decided to attempt simple fixation of the fracture with Kirschner wires (K‐wires).

## 2 Case Presentation

**FIGURE 1:**
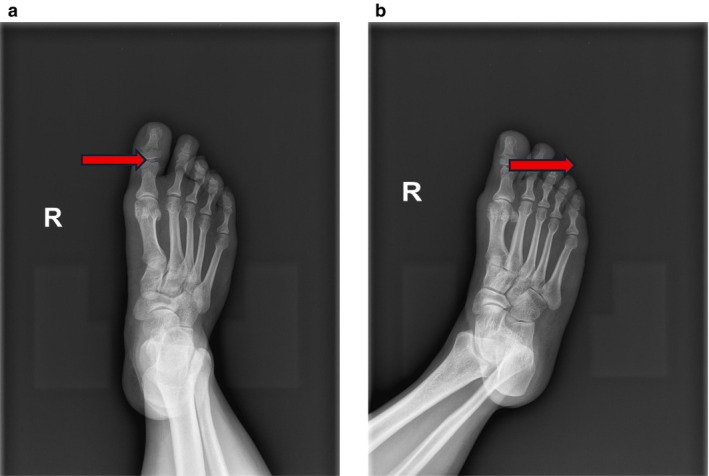
Preoperative radiograph of the right foot reveals a clear fracture of the distal phalanx and the presence of an intra‐articular sesamoid bone within the interphalangeal joint. The structure indicated by the red arrow corresponds to the sesamoid bone.

The patient was a 41‐year‐old female who worked as a construction worker. Who was admitted to the hospital due to “pain, swelling, and movement disorder of the right hallux for 3 h” caused by being crushed by an iron plate at the construction site. Clinical manifestations: Pain and swelling were present in the right hallux, accompanied by ecchymosis and cyanosis of the local skin and soft tissues, poor skin blood supply, subungual hematoma of the hallux, obvious bone crepitus palpable at the distal phalanx, limited functional activity of the hallux due to pain, and disturbance in walking and weight‐bearing functions. X‐ray examination revealed: Fracture of the distal phalanx of the right hallux with displacement of the fracture ends, and fracture of the proximal phalanx of the right hallux (Figure 1). After admission, relevant auxiliary examinations were improved, symptomatic treatment for detumescence was given, and after excluding surgical contraindications, closed reduction and K‐wire internal fixation for phalangeal fractures of the toe were performed under epidural anesthesia.

### 2.1 Surgical Procedure

The patient was placed in a supine position. After successful epidural anesthesia, the right lower extremity was disinfected with povidone‐iodine three times, and sterile drapes were routinely laid. Additionally, a drill hole was made on the nail plate of the right hallux to drain the subungual hematoma. The surgeon pinched the fracture ends with fingers, performed longitudinal traction reduction, and corrected lateral displacement, then selected a 1.2‐mm K‐wire to penetrate from the toe tip through the fracture line into the proximal and middle phalanges. Fluoroscopy confirmed that the position of the K‐wire was not ideal; anatomical reduction could not be achieved even after four attempts of manual reduction. We first considered that the entrapped sesamoid bone exerted a lever effect, affecting fracture reduction. Second, due to the severe contusion of the patient's skin and soft tissues, we were concerned that open reduction would increase the risk of skin infection and necrosis. Furthermore, the surgical consent form specified closed reduction and K‐wire internal fixation, so we did not arbitrarily change the surgical plan against the patient's will. After the last attempt of manual reduction, the K‐wire was inserted; fluoroscopy showed that the K‐wire passed through the articular fracture line, the fracture alignment was poor, and the fixation only reached a small amount of cortical bone of the fracture fragments of the middle and proximal phalanges. We worried that repeated attempts to insert the K‐wire would lead to fragmentation of the fracture fragments, so after confirming that the stability of the K‐wire was acceptable, we decided to end the operation. The tail end of the K‐wire was left about 5 mm outside the skin, and the wound was dressed with sterile gauze to complete the operation. The patient was discharged 3 days after the operation. Regular X‐ray reexaminations were performed; 6 weeks after the operation, there was no pain or discomfort at the fracture end, and the K‐wire was removed after clinical fracture union. At 3 months of postoperative follow‐up, the patient had returned to her hometown; telephone follow‐up showed that she had no pain or discomfort, could walk normally with the affected foot, and the function of the right foot was good.

Timeline of Key Clinical Events (As shown in Table 1).

**TABLE 1:** Timeline of key clinical events.

Time point	Clinical event
Day 0	The patient was admitted to hospital, and X‐ray examination was performed.
Day 7	Surgical intervention was performed.
Day 11	The patient was discharged in a stable clinical stable.
Day 30	Follow‐up radiography at the outpatient clinic revealed obvious callus formation at the fracture site.
Day 45	Kirschner wire was removed after radiographic confirmation of fracture union.

## 3 Discussion

**FIGURE 2:**
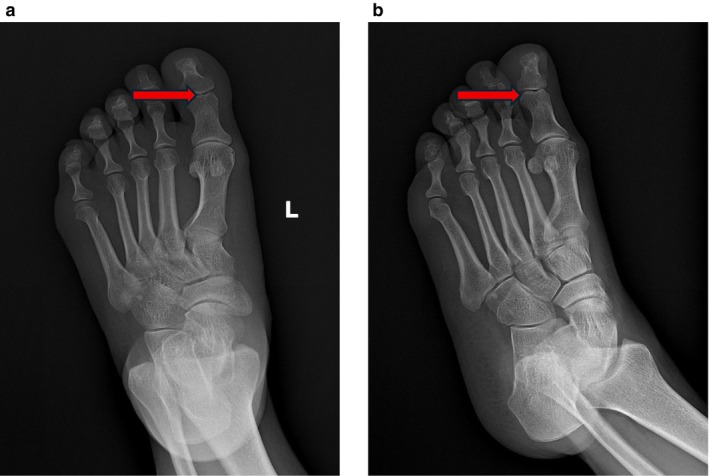
Preoperative radiograph of the left foot demonstrates the presence of a sesamoid bone within the interphalangeal joint. The structure indicated by the red arrow corresponds to this sesamoid bone. Based on these findings, the patient is considered to have congenital sesamoid impaction.

**FIGURE 3:**
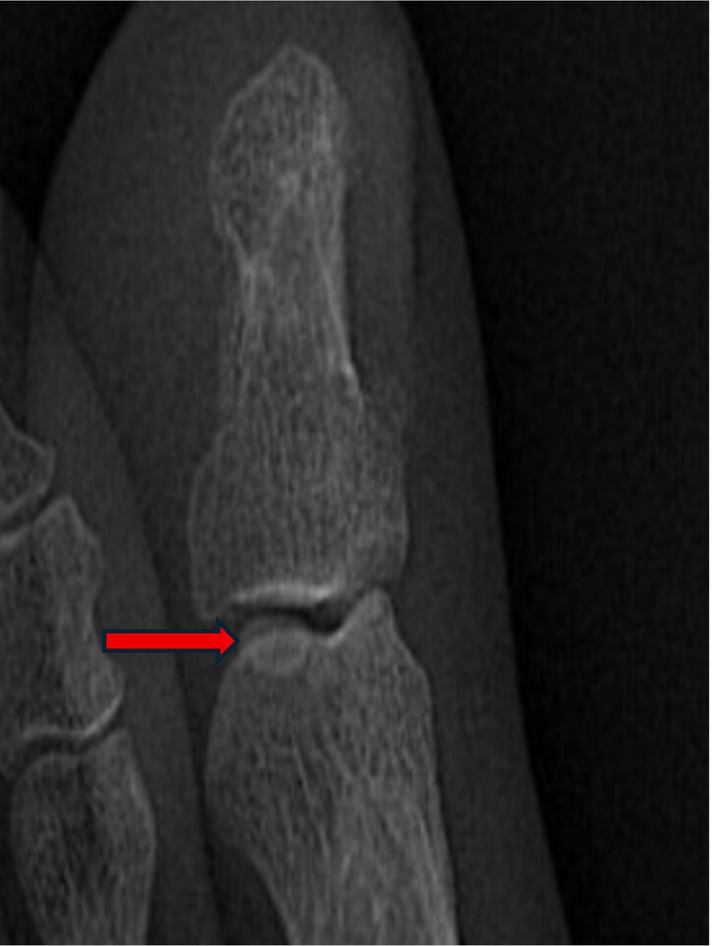
Magnified radiographic imaging of the left foot clearly reveals a sesamoid bone within the interphalangeal joint. The structure indicated by the red arrow corresponds to this sesamoid bone.

**FIGURE 4:**
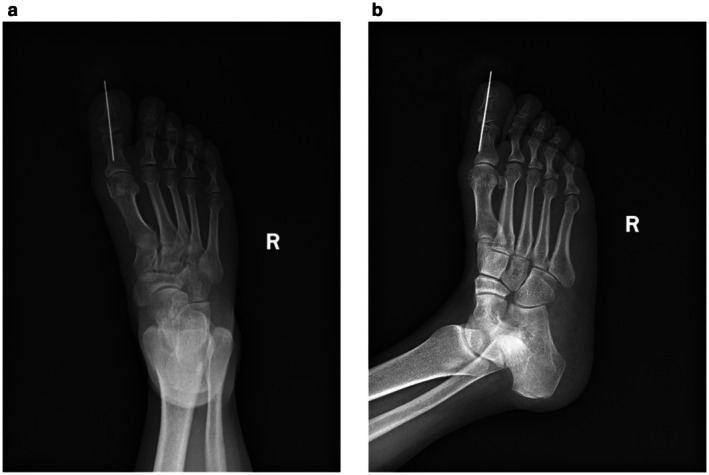
Radiographic evaluation of the right foot postoperatively revealed malalignment of the fracture due to impingement by a sesamoid bone.

**FIGURE 5:**
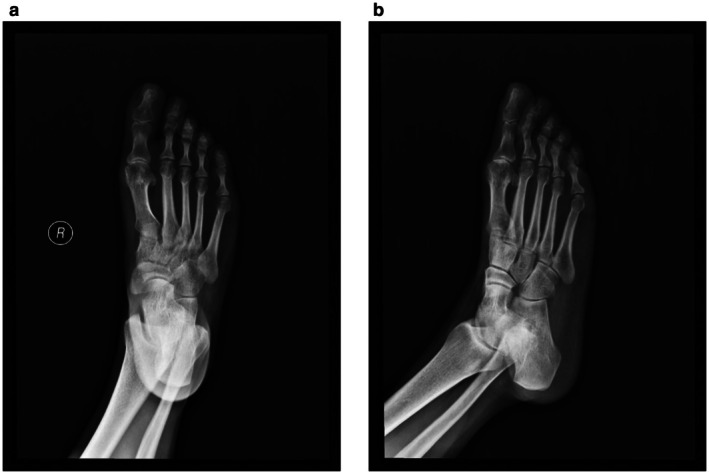
Radiographic imaging of the right foot obtained 8 weeks following removal of the Kirschner wires confirms complete fracture healing.

The sesamoid bones of the hallux of the foot are usually located on the plantar side of the first metatarsophalangeal joint. They are divided into the tibial sesamoid (larger) and the fibular sesamoid (smaller). These do not enter the joint cavity under normal physiological conditions. Their functions include tendon protection, mechanical advantage, and joint stability. Previous reports describe dorsal [1, 2, 3] and medial [4, 5] surgical approaches for phalangeal fracture reduction. The dorsal approach requires incision of the extensor tendon, while the medial approach offers better visualization [3]. Anatomical studies suggest the medial approach may be most suitable for intra‐articular locations [6], yet the dorsal approach is considered simpler and safer [7]. Some literature suggests the sesamoid excision [7, 8, 9] is unnecessary [10]. Shinsuke Takeda reported a case of closed reduction for interphalangeal joint dislocation [11]. However, our patient had a concomitant fracture. Given the sesamoid entrapment and the scarcity of reports on percutaneous reduction for such cases [12], reduction was challenging. We initially considered open surgery to remove the sesamoid before fixation. Open reduction is associated with great trauma, extensive soft tissue dissection, and a risk of incision complications. External fixators are mostly used for severely comminuted or open fractures; they have a relatively complex structure, are inconvenient for daily care and life, and are not suitable for this type of fracture. Considering that the patient's right foot was crushed by a heavy object, resulting in swelling, ecchymosis, cyanosis, and severe contusion of the skin of the right foot, as well as poor local skin and soft tissue blood supply, we were concerned that open reduction or other excessive operations would lead to an increased risk of skin necrosis and infection. In addition, both the articular surfaces of the distal phalanx and the proximal phalanx were fractured, and cross K‐wire fixation would increase the surgical risks, such as damage to the proximal phalanx fragments or fragmentation of the fracture fragments. After thorough discussion, we concluded that the surgical plan of opening the joint capsule for reduction and resecting the sesamoid bone would be associated with extensive soft tissue trauma, requiring disruption of the joint capsule and increasing unpredictable risks for the patient. Out of curiosity, we performed an X‐ray examination on the patient's left foot and found that there was also sesamoid entrapment in the interphalangeal joint of the left foot; the patient had sesamoid entrapment in the left foot as well, but with normal mobility (Figures 2 and 3). Following Woon's recommendation [12], we opted for K‐wire fixation of the distal phalanx of the toe and the joint (Figure 4). Percutaneous K‐wire fixation causes minimal interference to the blood supply around the fracture and soft tissues, which is more conducive to fracture healing, results in faster postoperative recovery, meets the patient's requirement of returning to work as soon as possible, and the K‐wire can be removed about 6 weeks later when the fracture heals (Figure 5), thus avoiding additional expenses for the patient. Intraoperatively, there was a risk of the K‐wire penetrating or fracturing the sesamoid, potentially fixing it within the joint. Therefore, repeated fluoroscopy was used to avoid capturing the sesamoid. While this precaution contributed to suboptimal fracture reduction and potential extensor tendon irritation [8], it was deemed less damaging than open tendon and capsular disruption.

### 3.1 Limitations

(1) Due to the limitations of China's medical insurance policy, more examinations could not be performed. We only performed X‐ray examinations on both feet and did not conduct more precise CT scans or other examinations to clarify the position of the sesamoid bones. (2) We simply speculated that the patient had congenital sesamoid entrapment only based on the presence of sesamoid bones on the X‐ray of the other foot and the patient's lack of a previous trauma history, which is not rigorous. However, there is currently no technical means to confirm that the entrapped sesamoid bones of this patient are congenital. (3) In addition, we did not strengthen the preoperative communication with the patient to fully consider the possible intraoperative accidents and formulate corresponding treatment measures. (4) Due to the limitations of the study design and follow‐up conditions, we did not record objective data such as the range of motion of the joints of both feet, nor compare the postoperative activity range of both feet and the recovery of postoperative pain to evaluate the postoperative functional recovery. We will further improve the relevant functional evaluation indicators in future studies.

## 4 Conclusion

We report a rare case of distal phalanx fracture of the foot with interphalangeal joint sesamoid entrapment. Frankly, although the sesamoid bone was not resected through an incision during the operation, which would have caused greater trauma, we do not consider this a successful operation due to the lack of anatomical reduction and the presence of certain fracture displacement. Combined with previously published relevant cases and the difficulties encountered in our actual surgical operation, the entrapment of the sesamoid bone during the operation provided a lever force, leading to difficulty in reduction. Although the patient had good fracture healing and satisfactory recovery of weight‐bearing walking function after the operation, we are more inclined to perform open reduction, resect the sesamoid bone, and then perform reduction and fixation. This surgical method can achieve more accurate fracture reduction and avoid long‐term pain caused by joint cartilage wear or even traumatic arthritis due to sesamoid entrapment in the later stage. Percutaneous K‐wire fixation can achieve a direct short‐term curative effect, but it has long‐term risks and limitations. Based on the difficulties encountered during the operation and postoperative reflection, we believe that open reduction with sesamoid bone resection is a better surgical option. Its advantages stem from fundamentally avoiding potential problems caused by nonanatomical reduction and providing a more reliable long‐term prognosis for the patient. We hope to report our unsuccessful case to provide better clinical ideas and references for the diagnosis and treatment of such fractures.

## Author Contributions

**Weihao Zhu:** writing – original draft. **Yan Cheng:** writing – original draft. **Xu Sun:** project administration.

## Funding

The authors have nothing to report.

## Consent

Written informed consent was obtained from the patient for publication of this case report and any accompanying images.

## Conflicts of Interest

The authors declare no conflicts of interest.

## Data Availability

The authors have nothing to report.
